# *Rickettsia mongolitimonae* Encephalitis, Southern France, 2018

**DOI:** 10.3201/eid2602.181667

**Published:** 2020-02

**Authors:** María Dolores Corbacho Loarte, Cléa Melenotte, Nadim Cassir, Serge Cammilleri, Philippe Dory-Lautrec, Didier Raoult, Philippe Parola

**Affiliations:** Hospital Universitario Severo Ochoa, Madrid, Spain (M.D. Corbacho Loarte);; Institut Hospitalo-Universitaire–Méditerranée Infection, Marseille, France (M.D. Corbacho Loarte, C. Melenotte, N. Cassir, D. Raoult, P. Parola);; Aix-Marseille University, Marseille (M.D. Corbacho Loarte, C. Melenotte, N. Cassir, D. Raoult, P. Parola);; Assistance Publique des Hôpitaux de Marseille, Marseille (C. Melenotte, N. Cassir, S. Cammilleri, P. Dory-Lautrec, D. Raoult, P. Parola)

**Keywords:** Rickettsia mongolitimonae, meningitis/encephalitis, tickborne rickettsioses, vector-borne infections, ticks, bacteria, France

## Abstract

We report a case of *Rickettsia sibirica mongolitimonae* infection, an emerging tickborne rickettsiosis, with associated encephalitis in a 66-year-old man. Diagnosis was rapidly confirmed by quantitative PCR obtained from an eschar swab sample. The patient was successfully treated with oral doxycycline.

In July 2018, a 66-year-old man was admitted to the emergency department in Marseille, France, because of fever (40°C) and confusion. His medical history included arterial hypertension controlled with amlodipine and dyslipidemia and coronary artery disease treated with pravastatin and aspirin. He lived in a rural area near Marseille and owned dogs, pigs, pheasants, pigeons, and chickens. In the hospital emergency department, he received acyclovir (1 g every 8 h), amoxicillin (4 g every 6 h), and ceftriaxone (3 g every 12 h) for suspected meningoencephalitis.

At admission to the infectious diseases department, he had a general maculopapular rash over his trunk, palms of his hands, and soles of his feet of 3 days’ duration (Figure, panel A). Blood pressure was 130/80 mm Hg. A 15-mm black eschar was noted on his right ankle, associated with rope-like lymphangitis (Figure, panel B). He had a 4/5 right corporal hemiparesis with hemisensory loss and right Babinski sign. Lumbar puncture results were unremarkable, and C-reactive protein was 65.4 mg/L (referent <3 mg/L). Oral doxycycline (300 mg 1×/d) was added to his drug regimen 3 days after symptom onset. Results of brain computed tomography scan were unremarkable. Magnetic resonance imaging showed multiple bilateral brain lesions compatible with acute encephalitis related to vasculitis (Figure, panel C). Positron emission tomographic scan showed cerebral cortical diffuse hypometabolism (Appendix Figure). Results of microbiological tests performed on cerebrospinal fluid and indirect immunofluorescence assay for spotted fever group (SFG) rickettsiae were negative. DNA obtained from eschar swab samples was positive by quantitative PCR for all SFG *Rickettsia* species (*gltA* and *ompA* genes) (*1*). Positive samples tested with species-specific *R. massiliae*, *R. conorii*, and *R. sibirica mongolitimonae* primers were positive for *R. sibirica mongolitimonae* (35 cycles quantification) (*1*). 

**Figure:**
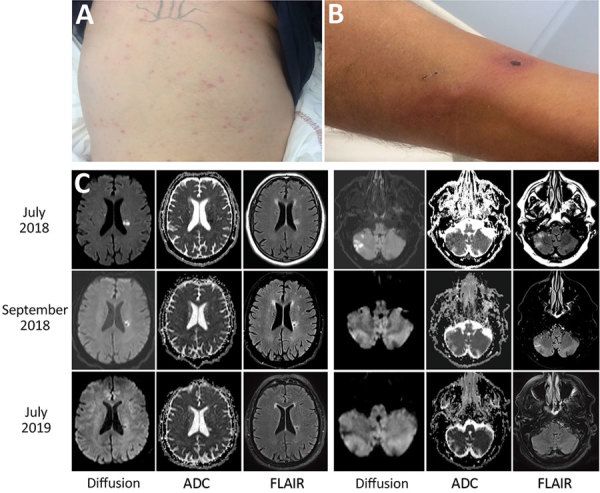
Clinical manifestations and cerebral magnetic resonance imaging of a 66-year-old man with *Rickettsia sibirica mongolitimonae*–associated encephalitis, southern France, 2018. A) Maculopapular rash. B) Black eschar and rope-like lymphangitis on the right leg. C) Magnetic resonance imaging with diffusion (B1000), ADC, and FLAIR. In July 2018, cytotoxic lesions were observed intra-axially and in the white matter of right cerebellar hemispheres with FLAIR hypersignal and with low ADC signal. In September 2018, these cytotoxic lesions regressed in diffusion with the appearance of a necrotic cavity facing the roof of the left lateral ventricle. In July 2019, disappearance of diffusion anomalies. Small necrotic cavity with after-effects on FLAIR and ADC signals. ADC, apparent diffusion coefficient; FLAIR, fluid-attenuated inversion recovery.

Oral doxycycline was continued for 10 days; other drugs were discontinued. The cutaneous lesions regressed at day 3, and neurologic symptoms progressively improved after administration of doxycycline. A low seroconversion for the SFG rickettsiae was observed (IgM 1:16; IgG 1:16) 3 weeks after symptom onset; at 7 weeks postinfection, serology became negative. 

One month after symptom onset, the patient had 4/5 muscular strength in his right leg. Magnetic resonance imaging performed at 7 weeks and 1 year after symptom onset showed cerebral sequlae lesions (Figure, panel C). At 1 year, the Babinski sign in the right foot persisted, but muscular testing was 5/5 with the exception of lifting the right foot, which was 4/5.

*R. sibirica mongolitimonae* infection is an emerging rickettsiosis; <40 human cases have been described. It is seasonal in France (spring and summer). It has been referred to as lymphangitis-associated rickettsiosis because of the typical rope-like lymphangitis sign (*2*). Other clinical signs include the classic triad of fever, rash, and eschar. SENLAT (scalp eschar and neck lymphadenopathy after tick bite) also has been reported (*3*). 

Most *R. sibirica mongolitimonae* infections have been reported in the Mediterranean area (France, Spain, Portugal, Greece, and Turkey), Africa (Algeria, Egypt, Cameroon, South Africa), and China (*4*,*5*). In Europe, vectors include the tick species *Hyalomma excavatum*, *H. marginatum*, *H. turanicum*, *Rhipicephalus pusillus*, *R. bursa*, and *Haemaphysalis parva* (*1*,*2*,*4*). *R. sibirica mongolitimonae* infection usually causes mild disease, but severe manifestations have been described, including retinal vasculitis, lethargy with hyponatremia, septic shock, myopericarditis, and acute renal failure (*2*,*6*).

Only *R. conorii conorii*, *R. rickettsii*, *R. japonica*, and *R. slovaca* have been associated with encephalitis in the literature (Appendix Table); no patients who had received doxycycline were reported to have died. Doxycycline has proven to be superior to chloramphenicol and ciprofloxacin in rickettsial infection and should be the treatment of choice for rickettsial-associated encephalitis (*1*,*7*).

SFG rickettsiosis can be diagnosed by serology, culture, or molecular assay on blood, skin biopsy, or eschar swab sample. Seroconversion generally appears in the second and third weeks of illness; culture is fastidious and performed only in expert laboratories. Molecular tools using eschar cutaneous swab samples appeared as the best method for detecting and identifying *Rickettsia* spp. (*1*). The sensitivity of this technique is comparable with that of rickettsial detection on skin biopsy samples using molecular tools. It is a noninvasive and nonpainful diagnostic method that can be performed easily where molecular facilities are available (*3*,*8*). 

The discrepancy observed in this case between PCR and serology has been reported in cases of *R. africae* infection, in which seroconversion is delayed (28 days for IgG and 25 days for IgM) and doxycycline treatment within 7 days after symptom onset prevents development of antibodies (*9*,*10*). In this patient, we observed very low serologic response 3 weeks after symptom onset, which might have been affected by the early administration of doxycycline. Moreover, the lack of serologic response observed here may be precisely related to the severity of the disease. The case we described illustrates the rapid efficacy of doxycycline to treat the severe neurologic consequences of rickettsial diseases, as well as the effectiveness and rapidity of the swab sample diagnostic test.

Appendix Additional information about *Rickettsia mongolitimonae* encephalitis.
