# Supplementary material for: Rickettsia mongolitimonae Encephalitis, Southern France, 2018
Source: Emerg Infect Dis. 2020 Feb;26(2):362–4. doi: 10.3201/eid2602.181667 (PMC6986838; doi:10.3201/eid2602.181667)
Supplement: Appendix — Additional information about Rickettsia mongolitimonae encephalitis. [file 18-1667-Techapp-s1.pdf]

Article DOI: https://doi.org/10.3201/eid2602.181667

# *Rickettsia mongolitimonae* Encephalitis, Southern France, 2018

## Appendix

**Appendix Table.** Encephalitis and *Rickettsia* spp.*

| | Epidemiologic data | | | | | Clinical manifestation | | | | | | |
|---|---|---|---|---|---|---|---|---|---|---|---|---|
| Case | Country | Rural | Date | Age/sex | Previous illness | General | Neurologic | CFS, CT scan, MRI | Diagnostic test | Treatment | Outcome | Ref |
| *Rickettsia conorii* | | | | | | | | | | | | |
| 1 | Italy | NA | 2013 Aug | 78 y/F | Arterial hypertension, type 2 diabetes, stroke | Fe + R + S, renal failure | Coma GCS3 | –CFS: meningitis –CT scan: normal –MRI: ND | IFA, ELISA | Chloramphenicol, levofloxacin, corticosteroids | Death | (*1*) |
| 2 | Portugal | Dogs | 2010 Sep | 59 y/M | None | Fe + R + S, myalgia | Headache, hemisensory loss | –CFS: no meningitis –CT scan: hypodensities frontal lobes and 1 lesion in left ventricle –MRI: multiple noncontrast enhancement, periventricular | IFA | Doxycycline 200 mg/12 h 4 d, doxycycline 100 mg/12 h 3 d | Hemisensory loss on the left | (*2*) |
| 3 | Morocco | Dogs | 2009 | 1.2 y/M | None | Fe + R + S | Convulsions | –CFS: meningitis –CT scan: normal –MRI: normal | IFA | Josamycin 12 h, corticosteroids | Bilateral decreased vision, seizures | (*3*) |
| 4 | Spain | Dogs | 2008 Jun | 66 y/M | Type 2 diabetes | Fe + R + S, myalgia, renal failure, thrombocytopenia | Headache, confusion, quadriplegia, aphasia | –CFS: no meningitis –CT scan: normal –MRI: diffuse lesions in frontal, parietal, occipital, corpus callosum, cerebellar peduncules, pons, and limbic area | IFA | Doxycycline 100 mg/12 h 10 d | Aphasia and right hemiplegia | (*4*) |
| 5 | Morocco | NA | 2004 Jul | 49 y/F | None | Fe + R | Headache, confusion | –CFS: meningitis –CT scan: normal –MRI: meningeal contrast enhancement and lesion in right frontal lobe | IFA | Ceftriaxone, ampicillin, doxycycline | Remission | (*5*) |

| Case | Epidemiologic data | | | Age/sex | Previous illness | Clinical manifestation | | CFS, CT scan, MRI | Diagnostic test | Treatment | Outcome | Ref |
|---|---|---|---|---|---|---|---|---|---|---|---|---|
| | Country | Rural | Date | | | General | Neurologic | | | | | |
| 6 | India | Dogs | 2009 | 4 y/F | None | Fe + R | Convulsions | –CFS: meningitis<br>–CT scan: normal<br>–MRI: ND | Weil-Felix, IFA | Chloramphenicol 75 mg/kg/d in 4 doses and doxycycline 2.2 mg/kg/12 h 10 d | Remission | (*6*) |
| 7 | Portugal | NA | 2003 Summer | 47 y/M | None | Fe + R + S, myalgia, renal failure, thrombo-cytopenia | Headache, convulsions, shock | –CFS: ND<br>–CT scan: normal<br>–MRI: ND | Inmuno-histochemistry | None | Death | (*7*) |
| 8 | Spain | NA | 2002 | 27 y/F | None | Fe, myalgia, nausea, abdominal pain | Headache, paresis on left side, convulsions | –CFS: no meningitis<br>–CT scan: normal<br>–MRI: contrast enhancement in the meninges and right parasylvian lesion | IFA | Valproate, clonazepam, ceftriaxon, acyclovir, doxycycline, phenobarbital, corticotherapy | Remission | (*8*) |
| 9 | Spain | None | 1999 Jul | 53 y/F | Adult celiac disease | Fe + R, myalgia, arthralgia, thrombo-cytopenia, hypotension | Confusion, paraplegia | –CFS: meningitis<br>–CT scan: normal<br>–MRI: lesions in left frontal lobe, cerebellar peduncules, and corpus callosum | IFA | Cefotaxime, acyclovir, isoniazid, rifampin, ethambutol, streptomycin, doxycycline, methylprednisolone | Paraplegia | (*9*) |
| 10 | Spain | NA | 1994 Summer | 65 y/M | Type II diabetes | Fe + R + S, myalgia | Confusion, incontinence, ataxia | –CFS: meningitis.<br>–CT scan: hypodensity in white matter<br>–MRI: ND | IFA | Doxycycline 100 mg/12 h 7 d | Remission | (*10*) |
| 11 | Morocco | NA | 1991 Jul | 6 y/M | None | Fe + R + S, arthralgia | Confusion, convulsions | –CFS: meningitis.<br>–CT scan: hypodensity in both internal capsules<br>–MRI: ND | IFA | Thiamphenicol 15 d, corticosteroids 21 d | Mutism and quadriplegia | (*11*) |
| 12 | Spain | NA | 1987 Summer | 77 y/F | Arterial hypertension, type 2 diabetes | Fe + R + S, myalgia, renal failure, thrombo-cytopenia. | Headache, stupor, shock | –CFS: ND<br>–CT scan: ND<br>–MRI: ND | IFA | Amoxicillin 11 d, tetracycline 1 d | Death | (*12*) |
| 13 | France | Dogs | 1984 Aug | 20 d/F | None | Fe + R + S, hepatomegaly, splenomegaly, thrombo-cytopenia | Inactivity, convulsion | –CFS: meningitis<br>–CT scan: ND<br>–MRI: ND | IFA | Ampicillin, gentamicin, spiramycin | Death | (*13*) |

| Case | Epidemiologic data: Country | Rural | Date | Age/sex | Previous illness | Clinical manifestation: General | Neurologic | CFS, CT scan, MRI | Diagnostic test | Treatment | Outcome | Ref |
|---|---|---|---|---|---|---|---|---|---|---|---|---|
| *Rickettsia japonica* | | | | | | | | | | | | |
| 14 | Japan | Brushy vegetation | 1992 Aug | 58 y/M | NA | Fe + R + S, nausea vomiting | Confusion | –CFS: meningitis –CT scan: Normal –MRI: NA | IFA | Cefmetazole, clindamycin, aztreonam, imipenem, minocycline | Remission | (*14*) |
| 15 | Japan | Bamboo tree | 1998 Apr | 55 y/M | NA | Fe + R, myalgia, nausea. vomiting, renal failure, shock | Headache, confusion, convulsions | – CFS: meningitis –CT scan: subdural hematoma –MRI: NA | IFA | Panipenem, acyclovir, minocycline | Death | (*15*) |
| 16 | Japan | NA | Aug | 77 y/M | NA | Fe + R + S | Confusion, convulsions | NA | IFA | Minocycline | Remission | (*15*) |
| 17 | Japan | NA | Aug | 78 y/M | NA | Fe + R, thrombo-cytopenia | NA | NA | IFA | Minocycline | Remission | (*15*) |
| *Rickettsia slovaca* | | | | | | | | | | | | |
| 18 | Slovakia | Garden | 1978 May | 33 y/F | None | Fe, myalgia, arthralgia, nausea, hepatomegaly | Headache, paresthesia, bradypsychia | –CFS: no meningitis –CT scan: ND –MRI: NA | IFA | Tetracycline, doxycycline | Minimum hemiparesia | (*16*) |
| *Rickettsia rickettsii* | | | | | | | | | | | | |
| 19 | USA | Dog, horses, chickens, parrots, rats, mice | 1993 Aug | 45 y/F | None | Fe + R + S, myalgia, thrombo-cytopenia | Confusion, convulsions, disorientation | –CFS: no meningitis –CT scan: white matter hypodensity and diffuse cerebral edema –MRI: multiple punctata area of increased signal through cerebral white matter | IFA | Imipenem, doxycycline | Remission | (*17*) |
| 20 | USA | NA | 1997 | 68 y/M | None | Fe + R, thrombo-cytopenia | Confusion, disorientation | –CFS: no meningitis –CT scan: normal –MRI: symmetrical zones of hyperintensity in periventricular white matter | IFA | Doxycycline | Attentional deficits, visuospatial difficulties and global impairment in memory | (*18*) |
| 21 | USA | Farm | 1999 | 7 y/M | None | Fe + R + S, myalgia, arthralgia | Headache, lethargy, convulsions | –CFS: meningitis –CT scan: NA –MRI: multiple foci of hyperintensity in white matter | IFA | Doxycycline, methylprednisolone | Deficit in short-term memory | (*19*) |
| 22 | USA | NA | 2016 Apr | 15 y/F | None | Fe + R | Convulsions | –CFS: NA –CT scan: NA | IFA | Doxycycline | Speech difficulty | (*20*) |

| | Epidemiologic data | | | Age/sex | Previous illness | Clinical manifestation | | | Diagnostic | | | |
|---|---|---|---|---|---|---|---|---|---|---|---|---|
| Case | Country | Rural | Date | | | General | Neurologic | CFS, CT scan, MRI | test | Treatment | Outcome | Ref |
| | | | | | | | | –MRI: multiple foci of hyperintensity in periventricular white matter | | | | |
| 23 | USA | Dog, cat | 1997 Jun | 43 y/F | None | Fe + R + S, occipital lymph node, thrombo-cytopenia, respiratory distress, acute myocardial infarction | Headache, confusion, aphasia, tetraparesia | –CFS: meningitis –CT scan: diffuse cerebral edema with cerebellar herniation –MRI: NA | IFA | Ceftriaxone, doxycycline, methylprednisolone | Death | (*21*) |
| 24 | USA | NA | 2015 Jun | 7 y/F | None | Fe + R + S, thrombo-cytopenia, renal failure, respiratory distress | Headache, disorientation, aphasia, dystonic posture | –CFS: no meningitis –CT scan: diffuse supratentorial attenuation –MRI: diffuse hyperintense lesion in white matter | IFA | Ceftriaxone, vancomycin, doxycycline | Aphasia | (*22*) |

*CSF, cerebrospinal fluid; CT, computed tomographic; Fe: fever, GCS: Glasgow coma score; CT: computed tomography ; MRI: magnetic resonance imaging ; NA: not available; IFA, immunofluorescence assay; NA, not available; ND, not done; R, rash; ref, reference; S, scar.

## References


1. Colomba C, Imburgia C, Trizzino M, Titone L. First case of Mediterranean spotted fever-associated rhabdomyolysis leading to fatal acute renal failure and encephalitis. Int J Infect Dis. 2014;26:12–3. PubMed http://dx.doi.org/10.1016/j.ijid.2014.01.024

2. Duque V, Ventura C, Seixas D, Barai A, Mendonça N, Martins J, et al. Mediterranean spotted fever and encephalitis: a case report and review of the literature. J Infect Chemother. 2012;18:105–8. PubMed http://dx.doi.org/10.1007/s10156-011-0295-1

3. Bougteba A, Basir A, Charradi N. Meningoencephalitis caused by *Rickettsia conorii* in a young infant [in French]. Rev Neurol (Paris). 2011;167:173–6. PubMed http://dx.doi.org/10.1016/j.neurol.2010.07.010

4. Aliaga L, Sánchez-Blázquez P, Rodríguez-Granger J, Sampedro A, Orozco M, Pastor J. Mediterranean spotted fever with encephalitis. J Med Microbiol. 2009;58:521–5. PubMed http://dx.doi.org/10.1099/jmm.0.004465-0

5. Demeester R, Claus M, Hildebrand M, Vlieghe E, Bottieau E. Diversity of life-threatening complications due to Mediterranean spotted fever in returning travelers. J Travel Med. 2010;17:100–4. PubMed http://dx.doi.org/10.1111/j.1708-8305.2009.00391.x

6. Tikare NV, Shahapur PR, Bidari LH, Mantur BG. Rickettsial meningoencephalitis in a child—a case report. J Trop Pediatr. 2010;56:198–200. PubMed http://dx.doi.org/10.1093/tropej/fmp065

7. Amaro M, Bacellar F, França A. Report of eight cases of fatal and severe Mediterranean spotted fever in Portugal. Ann N Y Acad Sci. 2003;990:331–43. PubMed http://dx.doi.org/10.1111/j.1749-6632.2003.tb07384.x

8. Parra-Martínez J, Sancho-Rieger J, Ortiz-Sánchez P, Peset V, Brocalero A, Castillo A, et al. Encephalitis caused by *Rickettsia conorii* without exanthema [in Spanish]. Rev Neurol. 2002;35:731–4. PubMed

9. Ezpeleta D, Muñoz-Blanco JL, Tabernero C, Giménez-Roldán S. Neurological complications of Mediterranean boutonneuse fever. Presentation of a case of acute encephalomeningomyelitis and review of the literature [in Spanish]. Neurologia. 1999;14:38–42. PubMed

10. Marcos Dolado A, Sánchez Portocarrero J, Jiménez Madridejo R, Pontes Navarro JC, García Urra D. Meningoencephalitis due to *Rickettsia conorii*. Etiopathological, clinical and diagnostic aspects [in Spanish]. Neurologia. 1994;9:72–5. PubMed

11. Benhammou. Fièvre Boutonneuse Méditerranéenne révélée par une atteinte neurologique grave. Archives Francaises Pédiatriques. 1991;48:635–6.

12. Walker GA, Masters KS, Shah DN, Anseth KS, Leinwand LA. Valvular myofibroblast activation by transforming growth factor-beta: implications for pathological extracellular matrix remodeling in heart valve disease. Circ Res. 2004;95:253–60. PubMed http://dx.doi.org/10.1161/01.RES.0000136520.07995.aa

13. Texier P, Rousselot JM, Quillerou D, Aufrant C, Robain D, Foucaud P. Fièvre boutonneuse méditerranéenne: à propos d'un cas mortel chez un nouveau-né. Archives Francaises Pédiatriques. 1984;41:51–3.

14. Araki M, Takatsuka K, Kawamura J, Kanno Y. Japanese spotted fever involving the central nervous system: two case reports and a literature review. J Clin Microbiol. 2002;40:3874–6. PubMed http://dx.doi.org/10.1128/JCM.40.10.3874-3876.2002

15. Kodama K, Senba T, Yamauchi H, Nomura T, Chikahira Y. Clinical study of Japanese spotted fever and its aggravating factors. J Infect Chemother. 2003;9:83–7. PubMed http://dx.doi.org/10.1007/s10156-002-0223-5

16. Mittermayer T, Brezina R, Urvölgyi J. First report of an infection with *Rickettsia slovaca*. Folia Parasitol (Praha). 1980;27:373–6. PubMed

17. Baganz MD, Dross PE, Reinhardt JA. Rocky Mountain spotted fever encephalitis: MR findings. AJNR Am J Neuroradiol. 1995;16(Suppl):919–22. PubMed

18. Bergeron JW, Braddom RL, Kaelin DL. Persisting impairment following Rocky Mountain spotted fever: a case report. Arch Phys Med Rehabil. 1997;78:1277–80. PubMed http://dx.doi.org/10.1016/S0003-9993(97)90345-2

19. Wei TY, Baumann RJ. Acute disseminated encephalomyelitis after Rocky Mountain spotted fever. Pediatr Neurol. 1999;21:503–5. PubMed http://dx.doi.org/10.1016/S0887-8994(99)00028-4

20. Wittler RR, Minns GO. An adolescent with fever, rash, and altered mental status. Clin Infect Dis. 2016;62:1610–1. PubMed http://dx.doi.org/10.1093/cid/ciw096

21. Samuels MA, Newell KL. Case records of the Massachusetts General Hospital. Weekly clinicopathological exercises. Case 32-1997. A 43-year-old woman with rapidly changing pulmonary infiltrates and markedly increased intracranial pressure. N Engl J Med. 1997;337:1149–56. PubMed http://dx.doi.org/10.1056/NEJM199710163371608

22. Sun LR, Huisman TAGM, Yeshokumar AK, Johnston MV. Ongoing cerebral vasculitis during treatment of Rocky Mountain spotted fever. Pediatr Neurol. 2015;53:434–8. PubMed http://dx.doi.org/10.1016/j.pediatrneurol.2015.07.003

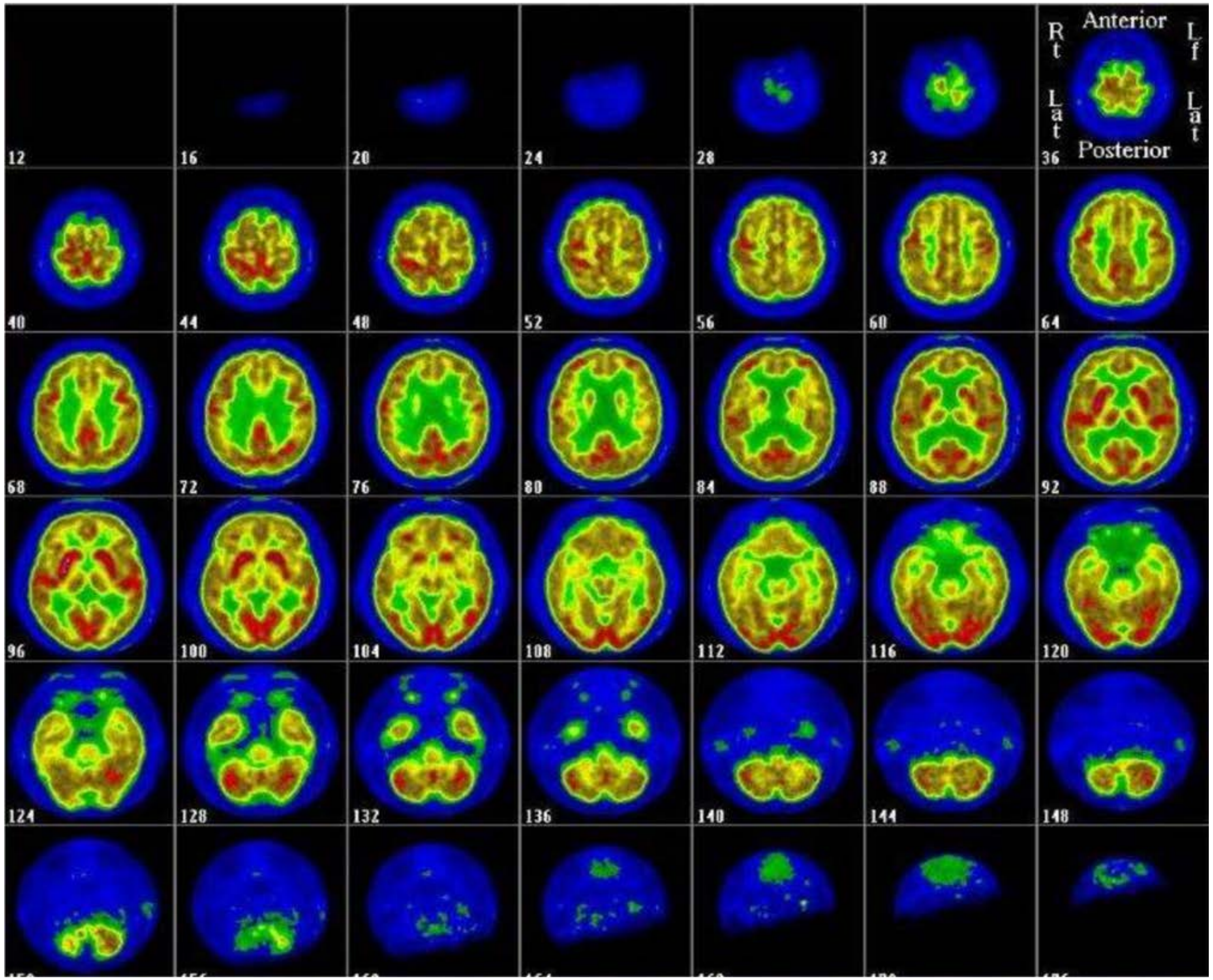


**Appendix Figure.** PET scan imaging showing a cerebral cortical diffuse hypometabolism.
